# Recent advances in the extraction, purification, structural-property correlations, and antiobesity mechanism of traditional Chinese medicine-derived polysaccharides: a review

**DOI:** 10.3389/fnut.2023.1341583

**Published:** 2024-01-17

**Authors:** Nannan Zhi, Xiangwei Chang, Xinrui Wang, Jian Guo, Juan Chen, Shuangying Gui

**Affiliations:** ^1^College of Pharmacy, Anhui University of Chinese Medicine, Hefei, China; ^2^Institute of Pharmaceutics, Anhui Academy of Chinese Medicine, Hefei, China; ^3^Anhui Province Key Laboratory of Pharmaceutical Preparation Technology and Application, Hefei, China; ^4^Engineering Technology Research Center of Modernized Pharmaceutics, Anhui Education Department (AUCM), Hefei, China; ^5^MOE-Anhui Joint Collaborative Innovation Center for Quality Improvement of Anhui Genuine Chinese Medicinal Materials, Hefei, China

**Keywords:** obesity, traditional Chinese medicine, polysaccharides, structural-property, antiobesity mechanism

## Abstract

Traditional Chinese medicine (TCM) has displayed preventive and therapeutic effects on many complex diseases. As natural biological macromolecules, TCM-derived antiobesogenic polysaccharides (TCMPOs) exhibit notable weight-loss effects and are seen to be a viable tactic in the fight against obesity. Current studies demonstrate that the antiobesity activity of TCMPOs is closely related to their structural characteristics, which could be affected by the extraction and purification methods. Therefore, the extraction, purification and structural-property correlations of TCMPOs were discussed. Investigation of the antiobesity mechanism of TCMPOs is also essential for their improved application. Herein, the possible antiobesity mechanisms of TCMPOs are systematically summarized: (1) modulation of appetite and satiety effects, (2) suppression of fat absorption and synthesis, (3) alteration of the gut microbiota and their metabolites, and (4) protection of intestinal barriers. This collated information could provide some insights and offer a new therapeutic approach for the management and prevention of obesity.

## Graphical Abstract

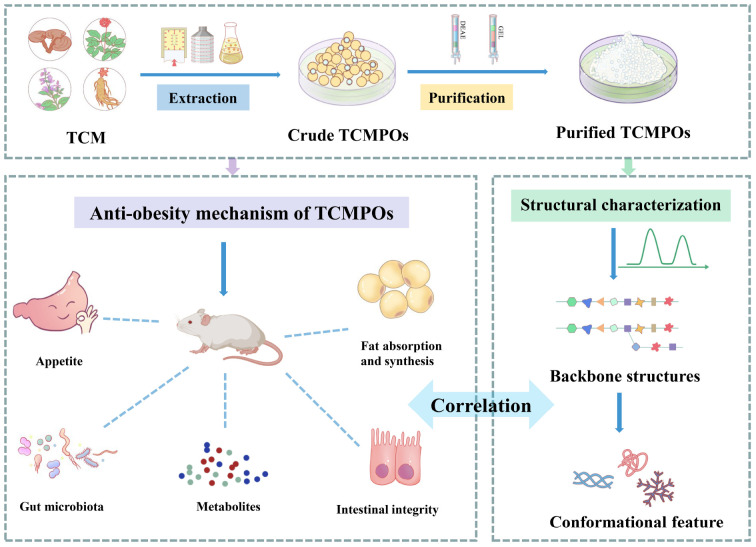

## Introduction

Obesity, one of the biggest challenges to public health worldwide to date, is also thought to be an important risk indicator for numerous other metabolic illnesses, such as hyperlipidemia, hypertension, myocardial infarction, stroke, type 2 diabetes as well as non-alcoholic fatty liver disorder, thereby contributing to a decline in quality of life and longevity (1–3). There has been a significant increase within the incidence of obesity globally, which is a major health risk to people and an important medical issue that needs to be resolved within various countries (2, 4, 5). Clinical strategies have been proposed to combat obesity mainly through drug administration and bariatric surgery (6, 7). Antiobesity drugs previously approved by the FDA, such as sibutramine, deoxyephedrine, carbamate and fenfluramine, have shown serious side effects, and some are no longer available or are unsuitable for long-term use (8–10). Bariatric surgeries, like Roux-en-Y gastric bypass (RYGB), gastric banding as well as sleeve gastrectomy, are confirmed to be more effective than drug therapy, but they have not been widely accepted due to their high cost, high risk, possible complications and sequelae (7, 11). Accordingly, great interest has been focused on exploring effective, healthy and low-risk alternative therapies to treat or alleviate obesity along with related metabolic disorders.

Traditional Chinese medicine (TCM) has been widely and safely applied within China for thousands of years due to its high efficiency and low side-effect, which is now becoming increasingly popular worldwide and is of vital significance to prevent disease or promote health (12, 13). As natural biological macromolecules, TCM-derived polysaccharides (TCMPs) have become highly attractive due to their remarkable biological functions and pharmacological properties, such as antioxidant, antidiabetic, antitumor, anti-inflammatory, anti-obesity, and immunomodulatory activities (5, 12, 14). In particular, the antiobesity activity of TCMPs has attracted the most attention. Multiple TCM-derived antiobesogenic polysaccharides (TCMPOs), such as *Zingiber striolatum* polysaccharides (13), *Rosa laevigata* polysaccharides (5), *Lycium barbarum* polysaccharides (14), *Cymodocea nodosa* polysaccharides (15), *Polygonatum cyrtonema* polysaccharides (16), *Laminaria japonica* polysaccharides (17), and *Ganoderma lucidum* polysaccharides (18), have been reported to have antiobesity effects. Considering different therapeutic leads, TCMPOs have two primary benefits over synthetic medications and bariatric procedures. Initially, the historical application of TCMs provides reliable empirical evidence for their safety. The antiobesity activity of naturally derived TCMPOs is more inclined to long-term effectiveness and safety and could be more acceptable by the public due to its distinct benefits, like affordable, few side effects as well as low risk (12, 16). Second, the antiobesity mechanism of TCMPOs is different from that of synthetic drugs; polysaccharides are hardly digestible and unabsorbable and can act on intestinal microbiota or intestinal hormones after oral administration, thereby producing potential antiobesity effects in the host (19, 20).

Numerous investigations have shown a close relationship between the structural properties for TCMPOs and their antiobesity efficacy, mainly involving the influence of molecular weights (Mw), backbone, conformational features and functional groups (12, 13, 21). Furthermore, the extraction and purification conditions of TCMPs, such as temperature, solvent and separation medium, could also have impacts on their compositions and structures, thus affecting their bioactivities (21–23). In addition, the antiobesity mechanism of TCMPOs has also become a research hotspot in recent years. Research shows that TCMPOs could exhibit antiobesity effects through multiple targets, such as regulating appetite, controlling the absorption and synthesis of fat, and modifying the intestinal flora (6, 24–26). Nevertheless, currently published reviews mostly focus on the separation, purification and biological activity of polysaccharides, there is no available review concerning the structural-property correlations and antiobesity mechanism of TCMPOs, despite having been sufficiently studied by many scholars. Therefore, our goal in this study is to methodically compile the results of contemporary research and to present a comprehensive understanding of the extraction, purification techniques, structural-property correlations and antiobesity mechanism of TCMPOs, thus drawing investigators’ attention to the application potential and utilization of TCMPOs.

## Extraction and purification of TCMPOS

### Extraction of TCMPOs

The most crucial step in the separation of TCMPOs is extraction. Table 1 depicts the methods and techniques used to extract, isolate and purify the polysaccharides from TCM. Table 1 illustrates that the traditional and most popular laboratory technique for producing TCMPOs is extract within hot or boiling water. In order to get useful TCMPOs yields, this approach often requires a high extraction temperature (60∼100°C), long duration (1∼24 h), high energy, a high water-to-material proportion, as well as several extraction stages. However, due to its relative conventionality, ease of control, and affordability, this approach is still widely utilized today. Additionally, to increase the extraction efficiency, a variety of extractant and supported techniques have been employed, including treatment with dilute acid (23, 27–30) or alkali (31, 32), high pressure (18, 33–35), high-power ultrasound (36, 37). Dilute acid or dilute alkali are capable of improving the amount extracted of polysaccharides, however several studies have shown that this method tends to damage the structure for TCMPOs which thus may further affect their biological activity (12, 38). According to earlier studies, the extraction yields of *Dictyophora indusiata* polysaccharides prepared using various extraction methods ranged from 5.62 to 6.48% (22). Compared with simple water extraction, microwave, ultrasonic and pressurized extraction take a shorter time and have higher extraction efficiencies (Table 1). However, in view of the above facts, the heat and extractant-assisted extraction method of TCMPOs is excessively conventional and has many limitations. Hence, the development of novel technology is imperative. For example, microwave extraction, which can generate high-frequency concussion and evenly penetrate the material to destroy the cell wall (39) and promote the extraction of intracellular polysaccharides (38) in a short time, is energy-efficient and could achieve a better yield. In addition, supercritical CO_2_ extraction is a productive extraction method that identifies unique characteristics of the extract at crucial stages (40). During this process, the use of appropriate entrainers or modifying agents such as ethanol and methanol (41, 42) improves the probability of obtaining TCMPOs.

**TABLE 1:** The source, geographical origins, extraction, isolation and purification of various TCMPOs.

No.	Source	Geographical origins	Compound name/ fractions	Extraction				Isolation and purification	Yield (%)	References
				Method of extraction	Time (Min)	Temperature (°C)	Material/Water ratio (g/mL)			
1*^a^*	*Cordyceps Sinensis*	Xi’an Ruilin Biotech Company	CSP	Water extraction	120	100	1:4	Ethanol precipitation; DEAE-52 ion exchange column; Sephadex G-100 gel column	–	(47, 58)
2*^b^*	*Wolfiporia cocos*	Yunnan, China	WIP	Alkali extraction (0.7 m NaOH)	240	100	1:18.8	Ethanol precipitation; Dialyzed; Water-soluble polysaccharides removed	39.8%	(31, 32)
3*^b^*	*Pueraria lobata* root	Bozhou, China, harvested in October	PLP	Water extraction	24*60	25	–	Dialyzed	24.3%	(166, 167)
4*^b^*	*Lycium barbarum*	–	LBP	Water extraction	30	100	1:10	Ethanol precipitation	–	(168, 169)
5*^a^*	*Sargassum fusiforme*	Qingdao, China, May	SFPs (Sf-1, Sf-2, Sf-3, Sf-3-1, Sf-A)	Hot-water and acid extraction	240	–	1:30	Sf-1 was eluted by water, Sf-2 was eluted by 0.5 M NaCl, Sf-3 was eluted by 2 M NaCl, Sf-3-1 was degraded from Sf-3, Sf-A was obtained from acid extraction of SFPs; DEAE Sepharose Fast Flow column, dialysis	–	(23, 27)
6*^b^*	*Sargassum fusiforme*	Qingdao, China, July	SFP	Ultrasound-assisted water extraction (400W)	150	90	1:20	Ethanol precipitation; Dialyzed	14.53%	(28, 37)
7*^b^*	*Sargassum fusiforme*	Qingdao, China, August	SfW	Hot water-extracted	60	–	–	Ethanol precipitation; Ultra-filtered	–	(29)
			SfA	Acid extraction	60	60	–	Ethanol precipitation; Dialyzed	–	
8*^b^*	*Sargassum fusiforme*	Wenzhou, China October	SFF	Acid extraction	360		1:20	Ethanol precipitation; Dialyzed	7.3%	(30, 170)
9*^b^*	*Rhodiola rosea*	Lin Chi Pharmacy of Zhenjiang, (Jiangsu, China)	RRP (RRP1, RRP2)	water extraction	120	90	1:10	RRP1 was eluted by the distilled water, RRP2 was eluted by 0.1M NaCl solution, DEAE-52 chromatographic column; Sephadex G-100 gel column	3.8%	(48)
10*^b^*	*Polygonatum cyrtonema*	Jiuhua Medicine Technology Co., Ltd. (Jiuhua, China)	PCP	Water extraction	120	90	1:10	Ethanol precipitation; Dialyzed	25.67%	(16)
11*^b^*	*Astragalus membranaceus*	–	APS	Water extraction	360	80	1:20	Ethanol precipitation	3.47%	(24)
12*^a^*	*Laminaria japonica*	Lianjiang, Fujian, China.	LJP61A	Water extraction	60	60	1:50	Ethanol precipitation; DEAE-cellulose column, Sephacryl S-500 column	–	(17, 137)
13*^a^*	*Laminaria japonica*	Lianjiang, Fujian, China.	LP (LP1, LP2, LP3)	Water extraction	60	60	1:50	Ethanol precipitation method at a different final concentration, 40% ethanol precipitation (LP1) 60% ethanol precipitation (LP2) 80% ethanol precipitation (LP3)	–	(49)
13*^b^*	*Ganoderma lucidum*	Chang Gung Biotechnology, China	WEGL (G1, G2, G3)	Pressurized water extraction	30	121	1:25	Ethanol precipitation; Membrane separation based on molecular weights	–	(18)
14*^b^*	*Portulaca oleracea* L.	Jilin Farmer’s Market	CPOP	Water extraction	540	80	1:20	Ethanol precipitation	9.6%	(60)
15*^a^*	*Ophiopogon japonicus*	Cixi, Zhejiang, China	MDG-1	Water extraction		95∼100	1:10	Ethanol precipitation; Sephadex G-25 column	–	(55, 171)
16*^a^*	*Ophiopogon japonicus*	Sichuan province, China	OJP	Water extraction (Three times)	90	100	1:4 1:4 1:2	Ethanol precipitation; DEAE-52 cellulose column	–	(53)
17*^a^*	*Liriope spicata*	Hubei province, China	LSP							
18*^a^*	*Liriope muscari*	Fujian province, China	LMP							
19*^a^*	*Mori fructus*	Buan-myeon, Gochang-gun, Jeollabuk-do, Korea	JS-MP-1	Water extraction	360	Room temperature	–	Ethanol precipitation and DEAE-cellulose ion exchange chromatography	0.09%	(46, 172)
20*^b^*	*Mori fructus*	Xinjiang, China	MFP	Water extraction	120	90	–	Ethanol precipitation	–	(54)
21*^a^*	*Morus Multicaulis Perr*	Shandong Agricultural University, Taian, China	MLP	Water extraction	240	100	–	Ethanol precipitation, ultrafiltration, DEAE Sepharose Fast Flow column, Sephadex G-100 column	0.13%	(45)
22*^b^*	*Dioscorea opposita* Thunb	Jiaozuo, Henan, China	CYP	Water extraction	120	90	–	Ethanol precipitation	–	(173)
23*^a^*	*Dendrobium officinale*	Yichang, Hubei, China	DOP	Water extraction	540	100	1:80	Ethanol precipitation, Sephadex G-50 column	–	(174)
24*^a^*	*Gastrodia rhizome*	Herbal Medicine Cooperative of Muju, Chonbuk, South Korea	GR-0	Water extraction	180	100	–	Ethanol precipitation, Dialyzed, DEAE-Sepharose CL-6B	–	(175)
25*^a^*	*Hirsutella sinensis*	–	H1, H2, H3, H4	Water extraction	30	121	1:25	Membrane separation based on molecular weights	–	(21)
26*^a^*	*Ginkgo biloba* leaf	Early November, Shandong, China	GBLP	Water extraction	18*60	80	1:30	Ethanol precipitation, Sephadex G-75 column	4.28%	(176)
27*^b^*	*Dictyophora indusiata*	Anhui Joy Lok Food Co., Ltd., Ningde, Fujian Province, China	DIP	Water extraction	120	100	1:30	Ethanol precipitation	13.2%	(162, 177)
28*^b^*	*Dictyophora indusiata*	Ya’an, China	DFP-H	Hot water extraction	150	95	1:30	Ethanol precipitation	5.52%	(22)
			DFP-M	Microwave assisted extraction (480w)	20	85	1:50		6.47%	
			DFP-U	Ultrasonic assisted extraction (650w)	15	Room temperature	1:22		6.48%	
			DFP-P	Pressurized assisted extraction (1.6 MPa)	30	55	1:30		6.39%	
29*^b^*	*Cyclocarya paliurus*	Zhangjiajie Nuokang Ecological Tea Co., Ltd., Hunan, China	CPP	Pressurized water extraction	45	78	–	–		(33, 34)
30*^b^*	*Gelidium amansii*	Keelung, China	GHE	Pressurized water extraction	20	121	1:20	–		(35)

*^a^*purified;

*^b^*crude; – not detect.

### Purification of TCMPOs

**FIGURE 1:**
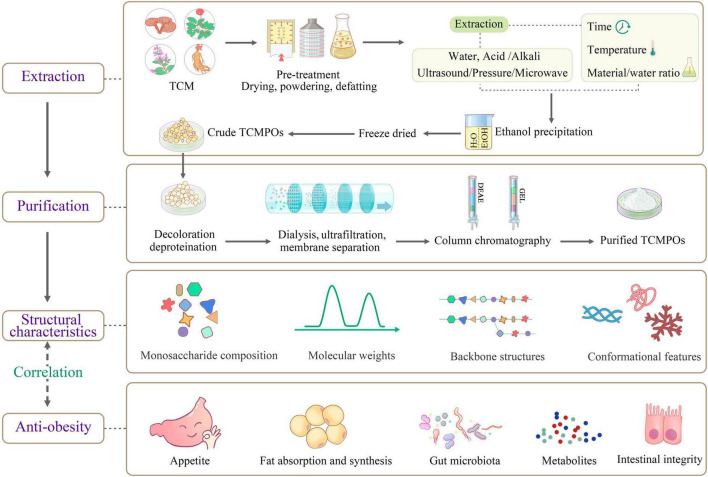
Schematic diagram of the extraction, purification, structural features and antiobesity mechanism of TCMPOs. TCM, traditional Chinese medicine; TCMPOs, traditional Chinese medicine-derived polysaccharides.

After the TCM water extracts are obtained, the isolation and purification process need to be first carried out by removing the non-polysaccharide components. The polysaccharides obtained through ethanol precipitation has been partly cleansed through deproteination as well as decoloration (38, 43, 44) before further being isolated and purified via dialysis (16), ultrafiltration (29, 45), membrane separation (18, 21), ion-exchange chromatography (46), gel filtration chromatograph (45, 47, 48), along with other methods. Figure 1 provides a schematic representation of the TCMPOs extraction and purification procedure. To obtain the pure fractions of TCMPOs, elution was carried out using the proper running buffers, then collection, concentration, dialysis, as well as lyophilization. For example, the water-extracted crude polysaccharides from *Sargassum fusiforme* were separated through a DEAE Sepharose Fast Flow column, as well as three polysaccharide fractions, namely, Sf-1, Sf-2, and Sf-3, were obtained from elution with water, 0.5 M NaCl, 2 M NaCl, respectively (23, 27). Wu et al. (21) fractionated the *Hirsutella sinensis* water extract into four fractions (H1, H2, H3 and H4) on the basis of their Mw through membrane separation. Zha et al. (49) fractionated the *L. japonica* water extract into the three fractions LP1, LP2, and LP3 using the ethanol precipitation method at ultimate concentrations of 40, 60, and 80%, correspondingly. Different separation and purification methods will lead to different TCMPOs components. However, some research suggests that most TCMPOs obtained by the present extraction, isolation and purification techniques are still crude products, and the quality of TCMPOs is difficult to control (12, 50, 51). First, the region of origin, the portions of the plant that are collected, the time of year it is harvested, and the technique of processing all affect how medicinally effective the TCM is (52). Second, non-template synthesized polysaccharides are never pure compounds regardless of how many procedures have been applied for purification (12). The acquired TCMPOs are always bound within either narrow or broad Mw ranges (12, 50). It is also worth noting that the extraction methods, solvent, equipment, personal operation method and so on differed from lab to lab. Therefore, special attention must be paid to the whole extraction, isolation and purification procedures to obtain high-quality TCMPOs.

## Physiochemical and structural features

The physiochemical and structural properties of TCMPOs, as shown in Figure 1, mainly including monosaccharide compositions, Mw, and backbone structures (configuration, type, position of glycosidic linkage, sequence of monosaccharide), as well as conformational features, have been investigated and identified by various chemical and instrumental analyses, such as acid hydrolysis, periodate oxidation analysis, methylation analysis, smith degradation, gas chromatography (GC) (49), gas chromatography–mass spectrometry (GC–MS) (53), ultraviolet (UV) detection (23, 32), ion chromatography (IC) (54), infrared spectroscopy (IR) (32), Fourier transform-infrared (FT-IR) spectroscopy (48), nuclear magnetic resonance (NMR) spectroscopy (55), high-performance liquid chromatography (HPLC) (56), high-performance size exclusion chromatography (HPSEC) (57), high-performance gel permeation chromatography (HPGPC) (58), scanning electron microscopy (SEM) (48), atomic force microscopy (AFM) and X-ray diffraction (XRD) (37, 48, 59). The structural characteristics of various TCMPOs, including their monosaccharide compositions, average Mw, backbone structures as well as conformational structures, are exhibited in Table 2, together with the sources, geographical origins and structural characterization methods.

**TABLE 2:** The physiochemical and structural features of various TCMPOs.

No.	Source	Geogra-phical origins	Obtained fractions	Monosaccharide composition										Molecular weight (Da)	Backbone structure	Conformational structure	Characterization method	References
				Glc	Gal	Man	Ara	Rha	GlcA	GalA	Xyl	GlcN	Fuc					
1	*Cordyceps Sinensis*	Xi’an Ruilin Biotech Company	CSP	90,262	526	317	264	184	151	–	–	–	–	6.486 × 10^4^	–	–	HPGPC; HPLC	(58)
2	*Wolfiporia cocos*	Yunnan province	WIP	–	–	–	–	–	–	–	–	–	–	4.031 × 10^5^ ∼4.486 × 10^6^	1,3-β-D-glucan	–	HPLC, IR, UV and NMR	(32)
3	*Sargassum fusiforme*	Qingdao, China, May	Sf-1	1	–	–	–	–	–	–	–	–		698.3/8.9 × 10^3^	–	–	HPSEC, HPLC, UV	(23)
			Sf-2	0.21	0.23	0.26	–	–	0.30	–	–	–	1	95.5/9.5 × 10^3^				
			Sf-3	–	0.24	–	–	–		–	–	–	1	2.295 × 10^5^				
			Sf-3-1	–	0.33	0.09	–	–	0.08	–	–	–	1	1.0 × 10^4^				
			Sf-A	1.26	0.42	0.10	–	–	0.05	–	–	–	1	46.5/5.1 × 10^3^				
4	*Sargassum fusiforme*	Qingdao, China, July	SFP	2.20	–	22.77	–	1.21	18.63	–	26.15	–	29.02	19.91/5.91 × 10^4^	–	No triple helix conformation	HPLC, UV, HPSEC, FT-IR, SEM, AMF	(28, 37)
5	*Sargassum fusiforme*	Qingdao, China, August	SfW	1.05	0.41	0.07	–	–	0.07	–	0.14	–	1	166/5.9 × 10^3^	–	–	HPSEC, NMR	(29)
			SfA	1.13	0.38	0.05	–	–	0.06	–	0.03	–	1	276/5.8 × 10^3^	–	–		
6	*Sargassum fusiforme*	Wenzhou, China	SFF	4.32	–	10.89	–	3.29	4.53	14.02	3.57	–	41.05	–	–	–	HPLC, FT-IR	(30)
7	*Rhodiola rosea*	Lin Chi Pharmacy of Zhenjiang, (Jiangsu, China)	RRP1	1	0.51	0.69	7.5	0.11	–	0.15	–	–	–	5.5 × 10^3^	1→6 or 1→ (50.63%), 1→2 or 1→4 (25.9%) and 1→3 (23.47%) glycosidic linkages	Possessed a triple helical conformation; a small lamellar or irregular dendritic structure	HPGPC, FT-IR, NMR, XRD, SEM, AFM	(48)
			RRP2	0.18	0.47	0.15	1	0.19	–	1.01	–	–	–	4.257 × 10^5^	1→6 or 1→ (23.5%), 1→2 or 1→4 (60.0%) and 1→3 (16.5%) glycosidic linkages	No triple helix conformation; a smooth surface topography with characteristic large wrinkles and drop-shaped bulges on the edges		
8	*Polygonatum cyrtonema*	Jiuhua Medicine Technology Co., Ltd. (Jiuhua, China)	PCP	15.09	37.30	19.45	–	18.78	–	15.62		–	–	5.1 × 10^3^	With both α- and β-configurations	–	HPGPC, FT-IR, NMR	(16)
9	*Astragalus membranaceus*	–	APS	70.55	3.61	–	23.39	1.6	–	–	0.84	–	–		–	–	GC	(178)
10	*Laminaria japonica*	Lianjiang, Fujian, China	LJP61A	1.85	2.92	1	–	–	–	–	–	–	–	1.96 × 10^6^	→3,6)-α-D-Manp-(1→, →4)-α-D-Manp-(1→, →4)-2-O-acetyl-β-D-Glcp-(→, →4)-β-D-Glcp-(1→, →6)-4-O-SO_3_-β-D-Galp-(1→, →6)-*upbeta*-D-Galp-(1→, →3)-β-D-Galp-(1→	–	GC, HPLC, NMR	(17, 137)
11	*Laminaria japonica*	Lianjiang, Fujian, China	LP1	0.47	1	1.46	2.63	–	–	–	–	–	–	1.73 × 10^7^, 8.18 × 10^6^, 1.01 × 10^6^	(1→6)-linked; (1→2)-/ (1→4)-linked (35%) and (1→3)-linked (5%) glycosyl bonds	–	HPLC, GC, FT-IR	(491)
			LP2	1.19	1.64	1.28	1	0.13	–	–	0.3	–	–	5.74 × 10^6^, 2.18 × 10^6^, 7.74 × 10^5^	(1→6)-linked, (1→3)- Linked (24%) and (1→2)-/(1→4)-linked (6%) glycosyl bonds	–		
			LP3	0.99	2.47	3.01	1	0.39	–	–	0.67	–	–	5.51 × 10^6^, 1.74 × 10^5^	(1→6)-linked, (1→2)-/(1→4)-linked (17%) and (1→3)-linked (59%) glycosyl bonds	–		
12	*Ganoderma lucidum*	Chang Gung Bio-technology, China	G1	26.3	16.9	47.5	2.9	2.5	–	–	–	–	2.9	>3 × 10^5^	–	–		(18)
			G2	–	–	–	–	–	–	–	–	–	–	1 × 10^4^∼3 × 10^5^	–	–		
			G3	–	–	–	–	–	–	–	–	–	–	<1 × 10^4^	–	–		
13	*Portulaca oleracea* L.	Jilin Farmer’s Market	CPOP	3.4	1	1.9	1.1	1	–	–	1.3	–	–	7.3 × 10^3^ (mainly), 1.19 × 10^4^, 9.3 × 10^4^	–	–	GC, HPSEC	(60)
14	*Ophiopogon japonicus*	Cixi, Zhejiang, China	MDG-1	–	–	–	–	–	–	–	–	–	–	3.4 × 10^3^	Fruf (2→1) with a branch of Fruf (2→6) Fruf (2→),α-d-Glc	–	NMR	(55)
15	*Ophiopogon japonicus*	Sichuan province, China	OJP	–	–	–	–	–	–	–	–	–	–	4.925 × 10^3^	Fruf-(2→,→2)-Fruf-(6→, →6)-Glcp-(1→ and →1, 2)-Fruf-(6→)	–	HPGPC, GC, GC-MS, NMR	(53)
16	*Liriope spicata*	Hubei province, China	LSP	–	–	–	–	–	–	–	–	–	–	4.742 × 10^3^				
17	*Liriope muscari*	Fujian province, China	LMP	–	–	–	–	–	–	–	–	–	–	4.138 × 10^3^				
18	*Mori fructus*	Buan-myeon, Gochang-gun, Jeollabuk-do, Korea	JS-MP-1	3.1	37,6	1.6	36.3	18.4	–	–	1.7	–	1.3	1.639 × 10^6^	α-Araf (terminal and 1,5- linked), β-Galp (1,3, 6-, 1, 6-, 1,4- and 1,3-linked), α-Rhap (1,2- and 1,2,4-linked), α-GalAp (terminal and 1,4-linked), terminal 4-O-methylated β-GlcpA	–	GC-MS, HPLC, NMR	(46, 172)
19	*Mori fructus*	Xinjiang, China	MFP	17.36	27.57	–	28.37	12.59	–	14.07	–	–	–	2.10 × 10^5^ (16.33%), 1.0 × 10^5^ (41.37%), 6.25 × 10^4^ (29.54%) and 2.08 × 10^4^ (12.76%)		–	IC, HPSEC	(54)
20	*Morus Multicaulis Perr*	Shandong Agricultural University, Taian, China	MLP	6.53	–	8.73	1	1.04	–	–	2.13	–	–	8 × 10^3^	β-glycosidic bond	–	HPLC, FT-IR, HPSEC	(45)
21	*Dendrobium officinale*	Yichang, Hubei, China	DOP	1	–	1.38	–	–	–	–	–	–	–	3.95 × 10^5^	–	–	HPGPC, GC	(174)
22	*Dendrobium officinale*		DOP	1	–	5.55	0.12	–	–	–	–	–	–	3.938 × 10^5^	–	–	FT-IR, GC, HPLC	(56, 179)
23	*Ginkgo biloba* leaf	Early November, Shandong, China	GBLP	9.39	32.21	20.82	6.71	14.76	–	16.11	–	–	–	1.2 × 10^4^	–	–	UV, HPGPC, HPLC	(176)
24	*Dictyophora indusiata*	Anhui Joy Lok Food Co., Ltd., Ningde, Fujian Province, China	DIP	59.84	12.95	23.25	0.17	0.043	1.014	–	0.36		1.58	–	–	–	HPLC	(161, 162)
25	*Dictyophora indusiata*	Ya’an, China	DFP-H	10.50	2.13	2.68		1.00	0.98	–	–	–	–	6.68 × 10^4^ ∼1.55 × 10^6^		–	–	(22)
			DFP-M	10.70	1.74	4.19		1.00	0.44	–	–	–	–	7.8 × 10^4^ ∼1.32 × 10^6^		–	–	
			DFP-U	8.83	1.24	2.68		1.00	0.46	–	–	–	–	5.91 × 10^4^ ∼1.47 × 10^6^		–	–	
			DFP-P	7.91	3.59	3.30		1.00	0.75	–	–	–	–	6.11 × 10^4^ ∼1.09 × 10^6^		–	–	
26	*Gelidium amansii*	Keelung, China	GHE	0.6	86	1.5		0.5	2.0	–	1.1	–	8.3	–	–	–	HPAEC-PAD	(35)

Glc, glucose; Gal, galactose; Man, mannose; Ara, arabinose; Rha, rhamnose; GlcA, glucuronic acid; GalA, galacturonic acid; Xyl, xylose; GlcN, glucosamine; Fuc, fucose. HPGPC, high-performance gel permeation chromatography; HPLC, high Performance Liquid Chromatography; IR, infrared Radiation; UV, ultraviolet; HPSEC, high-performance size exclusion chromatography; NMR, nuclear magnetic resonance; FT-IR, Fourier transform-infrared; XRD, X-ray diffraction; SEM, scanning electron microscopy; AFM, atomic force microscopy; HPAEC-PAD, high performance anion exchange chromatography-pulsed amperometric detector; GC, gas chromatography; GC-MS, gas chromatography–mass spectrometry; IC, ion chromatography; – not detect.

### Monosaccharide composition and proportion

Most often, the analysis of monosaccharide composition and proportion demands cleavage for glycosidic bonds via acid hydrolysis, followed by derivatization, detection along with measurement through HPLC, GC, or GC/MS (38, 43, 44, 57, 60, 61). In the process of derivatization, 1-phenyl-3-methyl-5-pyrazolone (PMP) is one of the best precolumn derivatization reagents in the determination of monosaccharides because its derivatives are not easy to cleave and generate isomeric peaks (48, 62). It is noteworthy that high-performance anion-exchange chromatography with pulsed amperometric detection (HPAEC-PAD) was increasingly used by investigators to detect the composition of monosaccharides because there is no need for derivatization (35, 63).

As shown in Table 2, most TCMPOs are mainly composed of glucose (Glc), galactose (Gal), mannose (Man), arabinose (Ara), rhamnose (Rha), glucuronic acid (GlcA), galacturonic acid (GalA), xylose (Xyl), and fucose (Fuc) with different molar ratios. It is noteworthy that *G. lucidum* polysaccharides were detected to contain glucosamine (GlcN) with a percentage of 1.1% (18). The diversity of monosaccharide composition and their molar ratio is associated with the TCM sources, geographical origins and harvest season. For example, monosaccharide composition differences were discovered for *S. fusiforme* harvested in Qingdao, Wenzhou, separately (27, 37). In addition, different monosaccharides were detected in the polysaccharides of *S. fusiforme* that were harvested in Qingdao in May, July, and August. The polysaccharides of *S. fusiforme* harvested in July had the highest content of Xyl and Rha (23, 28, 29, 37). The differences in monosaccharide composition in polysaccharide fractions are also closely relevant to the extraction and separation methods. For instance, DFP-H, DFP-M, DFP-U as well as DFP-P were obtained from *D. indusiata* with different extraction methods and possessed similar monosaccharide compositions but exhibited different molar ratios of constituent monosaccharides (22). LP1, LP2 along with LP3 were extracted and partitioned from *L. japonica* by the ethanol precipitation technique with various final concentrations. The dominant monosaccharides of LP1 were Ara, Man, Gal and Glu at a molar proportion for 2.63:1.46:1:0.47, whereas LP2 as well as LP3 comprised a total made up of Rha, Ara, Xyl, Man, Gal and Glu at molar proportions for 0.13:1:0.3:1.28:1.64:1.19 as well as 0.39:1:0.67:3.01:2.47:0.99, correspondingly (49).

### Average molecular weights

Multiple analytical techniques, including osmometry, viscosity measurement, sedimentation, GPC, HPGPC, HPSEC, and HPLC, are employed to determine the Mw distribution of TCMPOs, of which HPGPC and HPSEC are the most commonly utilized methods (38, 39, 43, 44, 64). As mentioned above, polysaccharides are never pure compounds regardless of how many procedures have been applied for purification. The acquired TCMPOs are always within either narrow or broad Mw ranges (12). Therefore, only the average Mw of TCMPOs can be detected. In addition, the average Mw mainly depend on the separation method, such as the different final ethanol concentrations (49), running buffers (23, 27, 48), separation membranes (18). Different Mw ranging from ∼10^3^ to ∼10^7^ Da were discovered under a variety of experimental conditions and source materials, which are demonstrated in Tables 1, 2.

### Backbone structures

The core of backbone structural analysis of TCMPOs is the sequence of monosaccharides and the configuration, type as well as location of glycosidic linkage. Generally, the priority is to investigate the backbone of polysaccharides with its branch link sites, the ring type (pyran or furan), the linkage sequence and the absolute configuration (D- or L-type) of monosaccharide residues, the α- or β-isomeric form of each glycosidic bond and feature groups (sulfate group, acetyl group, phosphate group, methyl group and so on) that may connect (12, 20, 65). Due to the complex structure of polysaccharides, the degradation (acid hydrolysis, periodate oxidation, smith degradation, etc.) of polysaccharides is usually performed first, followed by instrumental analysis methods (FT-IR, NMR, GC–MS, ion mobility-mass spectrometry, etc.) to obtain structural information (12, 48, 66, 67).

Studies have shown that the different fractions obtained from the same TCM may share the same glycosyl linkages, while the percentage of each glycosyl bond type varies significantly. For example, a structural investigation of two polysaccharide fractions (RRP1 and RRP2) from *Rhodiola rosea* indicated that RRP1 consisted of 1→6 or 1→ (50.63%), 1→2 or 1→4 (25.9%) as well as 1→3 (23.47%) glycosidic connections, while RRP2 consisted of 1→6 or 1→ (23.5%), 1→2 or 1→4 (60.0%) and 1→3 (16.5%) glycosidic connections (48). Furthermore, there were three polysaccharide fractions (LP1, LP2 and RRP2) from *L. japonica*, among which LP1 contained non-reducing terminal residues and (1→6)-linked (60%), (1→2)-/(1→4)-linked (35%) and (1→3)-linked (5%) glycosyl bonds, LP2 contained non-reducing terminal residues and (1→6)-linked (70%), (1→3)-linked (24%) and (1→2)-/(1→4)-linked (6%) glycosyl bonds, and LP3 contained non-reducing terminal residues and (1→6)-linked (24%), (1→3)-linked (59%) and (1→2)-/(1→4)-linked (17%) glycosyl bonds (49). Three kinds of maidong polysaccharides were examined for their structures, and these polysaccharides were all composed of Fruf-(2→,→2)-Fruf-(6→, →6)-Glcp-(1→ and →1, 2)-Fruf-(6→with molar ratios of 5.0:18.2:1.0:5.3 (*Liriope spicata*, LSP), 6.8:15.8:1.0:5.8 (*Ophiopogon japonicus*, OJP), as well as 8.3:12.3:1.0:3.9 (*Liriope muscari*, LMP) (53). In addition, the extraction and isolation method of TCMPOs may not affect their main chain structure. For instance, DFP-H, DFP-M, DFP-U, and DFP-P were obtained from *D. indusiata* with different extraction techniques, although variations in the kinds of glycosidic linkages and polysaccharide structures were not noticed (22). The structural characterization of TCMPOs is shown in Table 2, which indicates that most of the researchers actually did not further explore the structure of TCMPOs. The structure of TCMPOs is tightly related to biological activities (12, 57, 67), hence, it is particularly important to focus research on primary structure identification in the future.

### Conformational feature

Generally speaking, polysaccharides within aqueous solutions show a variety of chain conformations, including aggregates, random coils, as well as distinct helical forms (single, double, and triple helices) (43, 68–70). Some of the advanced technologies have been used to investigate the conformational features of polysaccharides, such as HPLC-static light scattering (HPLC-SLS) (70), HPLC-dynamic light scattering (HPLC-DLS) (70), viscosity test using the hypothesis of diluted polymer solutions (69), the Congo red test (28, 48), differential scanning calorimetry (DSC) (19), atomic force microscopy (AFM) (28, 43), circular dichroism (CD) (57, 69), fluorescence correlation spectroscopy (FCS) (69), transmission electron microscopy (TEM) (71), scanning electron microscopy (SEM) (28), and NMR spectroscopy (19, 72). For instance, research was done on the senior structures of two distinct polysaccharide fractions (RRP1 and RRP2) obtained from *R. rosea*, which were eluted using distilled water as well as 0.1 M NaCl solution, correspondingly (48), RRP1 was found to have a triple-helical conformation by the Congo red test, whereas RRP2 did not. RRP1’s surface included a modest lamellar or irregular dendritic structure, according to SEM tests, while RRP2’s surface topography was smooth and featured big wrinkles and drop-shaped bulges on the margins. AFM suggested that partial aggregation was observed in the polysaccharide chains of RRP1, and RRP2 had no molecular aggregations. These findings showed that the different elution solvents may have an impact on the advanced structure of TCMPOs. As shown in Table 2, only very few of the researchers have studied the senior structures of TCMPOs. The complexity and polymorphism of polysaccharides unquestionably bring major obstacles to their higher structural identification. Since the superior structures of polysaccharides may be more related to their bioactivities than to their primary structures (12, 51), further research and confirmation still need to be conducted in the future. Moreover, future technology development needs to concentrate on characterizing the high-level structural dynamic changes of polysaccharides (69).

## Correlation between the structure and antiobesity effects of TCMPOS

Few previous studies regarding the correlations between structure and antiobesity effects of various TCMPOs were stated, and it is not simple to connect the structures of TCMPOs with their antiobesity effects. Nevertheless, as shown in Figure 1, some correlations can be inferred as follows.

### Effect of molecular weight

It is generally accepted that the biological activities for polysaccharides are closely correlated with their Mw. According to related reports, TCMPOs with relatively high Mw usually demonstrate greater antiobesogenic activity than those with lower Mw. For example, varied Mw polysaccharides from *H. sinensis* oral administration to high fat diet (HFD)-induced obese rats, the high-Mw polysaccharides in fraction H1 (>300 kDa) showed greater antiobesogenic effects than the low-Mw polysaccharides in fraction H2 (10–300 kDa) as well as H3 (<10 kDa) (21). Administration of fraction H1 decreased body weight by ∼50% after 12 weeks and reduced the visceral fat and homeostatic model assessment-insulin resistance (HOMA-IR) index contrasted to the HFD control group, whereas fractions H2 and H3 only reduced the visceral fat pad mass and produced no influence on body weight or HOMA-IR index. In another study, the high-Mw polysaccharide PSF (≥100 kDa) from *Polygonatum kingianum* displayed better regulation of lipid metabolism via enhancing insulin sensitivity and more tightly controlling the make-up and activities of the gut microbiota (73). Comparable outcomes were also observed within the investigation of *G. lucidum* polysaccharide (>300 kDa) (18) and *Pseudostellaria heterophylla* polysaccharide (50∼210 kDa) (74).

Nevertheless, the association between the Mw of TCMPOs and their antiobesogenic activity is not simply a positive or negative correlation, and there are special cases that put forward different views (22, 75, 76). For instance, the antiobesity effects of four polysaccharides (DFP-H, DFP-M, DFP-U, and DFP-P) isolated from *D. indusiata* were investigated. The Mw size of the four polysaccharides was arranged as follows: DFP-H > DFP-P > DFP-M > DFP-U, whereas DFP-P showed the highest cholesterol/bile acids (BAs) attaching abilities as well as strongest inhibiting efficiency upon pancreatic lipase (22). Furthermore, it is worth noting that both DFP-P as well as DFP-H displayed significantly greater *in vitro* attaching characteristics, which includes fat, cholesterol as well as BAs, and lipase inhibiting properties compared to DFP-M and DFP-U, which may be because the relatively high Mw could reduce its water solubility, resulting in a decrease in hydrophilicity, thus enhancing the affinity for fat/cholesterol by increasing its surface aggregation (22, 76). The aforementioned data suggests that polysaccharides’ antiobesity effect is nevertheless enhanced by a reasonably high Mw. This may be explained by the fact that polysaccharides’ structural stability can only be preserved by a sufficiently high Mw (63). However, based on previous studies, maintaining a conformation with high biological activity of polysaccharides is not negatively impacted by either an excessive or modest Mw (77, 78), and the correlation between Mw and the antiobesogenic activity of TCMPOs should be further investigated.

### Effect of the backbone and conformational features

The biofunctional activities of TCMPOs could be affected by their backbones and conformational properties. Prior studies have demonstrated that the structural properties of polysaccharides, including types of glycosidic bonds, monosaccharide compositions and molar ratios, degrees of branching (DBs) in the backbone, are closely related to their biological activity (59, 79, 80). Studies have found that β-glycosidic linkages usually exhibit stronger antiobesity activity than α-glycosidic linkages, causing the α-amylase in human small intestine which is specific for α-glycosidic linkages (55, 75). Polysaccharides rich in β-glycosidic linkages can remain undigested and are sequentially fermented by colonic microflora, thus playing a relatively important role in weight loss (55). DFP-H, DFP-M, DFP-U, and DFP-P obtained from *D. indusiata* possessed similar monosaccharide compositions but exhibited different molar ratios. Findings indicated that DFP-P and DFP-H were better at binding fat than DFP-U and DFP-M, which might be related to the higher ratios of galactose and glucose (22). Two polysaccharide components (TSP-2 and TSP-1) were extracted through *Toona sinensis* leaves. TSP-1 demonstrated more hepatoprotective action than TSP-2, which may be related to TSP-1’s higher galactose and glucose levels as well as its higher branching residue concentration (81). It is worth mentioning that the DB in the backbone is not positively correlated with activity; only the suitable DB is advantageous to the arrangement of the triple helical configuration (79).

The conformational features (spherical, random coil, double-helix, triple-helix, worm-like, rod-like) of polysaccharides may be more related to their bioactivities than the primary structures. Polysaccharides with triple-helix conformations exhibit more potent biological effects, including anti-inflammatory, antidiabetic and immunomodulatory effects, than those with other structures, and some of them, such as lentinan and schizophyllan, have been used in clinical therapeutics (70, 82). As previously mentioned, two polysaccharide fractions (RRP1 and RRP2) isolated through *R. rosea* were identified with different conformational features, and subsequent research demonstrated that the triple-helix structure enabled fraction RRP1 to exhibit more prominent antioxidant and hepatoprotective properties than RRP2 (48). Although the above structure-bioactivity interaction analyses of TCMPOs were not aimed at understanding their antiobesity activities, the anti-inflammation, antioxidant and hepatoprotective activities were considered as the potential requirements for the treatment of obesity (83, 84). It can be inferred that the conformational features of TCMPOs will also affect their antiobesity activity.

### Effect of the functional groups

The functional group contents of polysaccharides are also linked to their bioactivities, like uronic acid, sulfuric acid and aminohexose (38, 85). Uronic acid residues, in particular, have the ability to modify the physiochemical characteristics and solubility of the corresponding polysaccharide conjugates, hence augmenting their biological activities. In a recent study, five fractions (MFPs-30-60, MFPs-30-80, MFPs-90-40, MFPs-90-60 as well as MFPs-90-80) had been purified from *Fructus Mori*, and *in vitro* studies revealed that MFPs-90-40, MFPs-90-60, and MFPs-30-60 showed a better capacity to bind BAs than MFPs-90-80 and MFPs-30-80, which was attributed to the increased uronic acid concentration and indicated that the uronic acid content was a significant signal representing the polysaccharide fractions’ hypolipidemic effects (86). In addition to the functional groups originally existing in polysaccharides, structural modifications, such as sulfation, acetylation, deacetylation, phosphorylation, acetylation, hydroxy-methylation, selenylation as well as complexation with zinc or iron, are of great significance to the physical and biological activities of polysaccharides. For example, the sulfation of *G. lucidum* polysaccharide (GLP) improved its antioxidant activities and BA-binding capacities (87). According to a different study, selenium modification may strengthen Chinese angelica polysaccharide’s hepatoprotective as well as antioxidant properties (88). Overall, the structure-bioactivity analyses of TCMPOs should be strengthened to better clarify the structural basis of their antiobesogenic activity.

## Antiobesity mechanism of TCMPOS

### Modulation of appetite and the satiety effect

**FIGURE 2:**
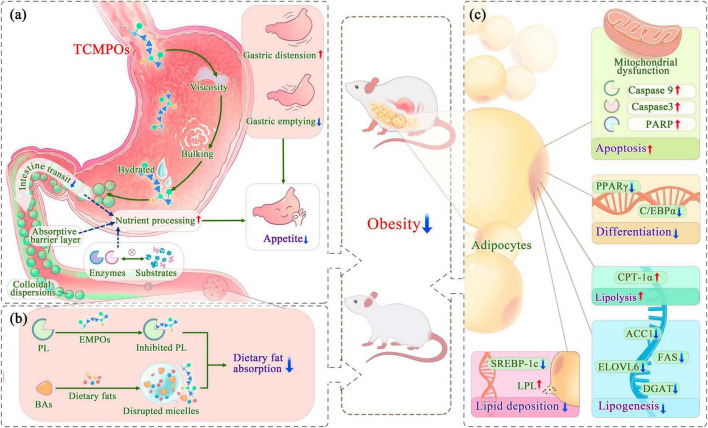
Antiobesity mechanism of TCMPOs based on the modulation of appetite and the regulation of fat absorption and synthesis. **(a)** TCMPOs modulate appetite and satiety effect. **(b)** TCMPOs regulate the digestion and absorption of exogenous lipids. **(c)** TCMPOs combat obesity by weakening the synthesis of endogenous lipids. TCMPOs, Traditional Chinese medicine-derived polysaccharides; PL, Pancreatic lipase; BAs, bile acids; PARP, poly (ADP-ribose) polymerase; PPARγ, peroxisome proliferator-activated receptor γ; C/EBPα, CCAAT/enhancer binding protein α; LPL, lipoprotein lipase; SREBP-1c, sterol regulatory element binding protein-1c; CPT-1α, carnitine palmitoyltransferase-1 alpha; ACC1, acetyl-CoA carboxylase 1; FAS, fatty acid synthase; ELOVL 6, long-chain elongase; DGAT, diacylglycerol acyltransferase.

Polysaccharides intake mainly regulates appetite and satiety through physical effects and chemical humoral stimulation effects (6), hence limiting the amount of food and energy consumed to stop the development of obesity. As shown in Figure 2, with bulking and viscosity-producing capabilities, TCMPOs could contribute to postprandial satiety by inducing gastric distension and delaying gastric emptying (89). Moreover, the appropriate ingestion of TCMPOs (such as *Amorphophallus konjac* polysaccharides) that, when hydrated, form viscous colloidal dispersions impacts several aspects of gastrointestinal (GI) function, including slowing down the transit of the small intestine (90, 91), inhibiting dietary enzymes from adhering to their substrates (especially fats and carbohydrates) (89), and creating an absorbent barrier layer within the small intestine by interacting with the mucosa (89, 91–93). This prolongation of the intestinal phase of nutrient processing and absorption may enhance satiety and help regulate food intake (89, 94).

More significantly, TCMPOs have the ability to control the release of gut hormones as well as peptides that influence appetite along with food intake, including leptin, adiponectin, glucagon-like peptide 1 (GLP-1), and peptide YY (PYY). A study demonstrated that *Holothuria leucospilota* polysaccharide (HLP) supplement outcomes with an amelioration in the levels of leptin and adiponectin, with the levels of leptin concentrations in the typical range exhibit benefits for preventing appetite, improving fatty acid oxidation, as well as decreasing body fat, but excess levels of leptin could lead to insulin resistance (95). Most notably, HLP effectively reversed the overexpression of hepatic acetyl-CoA carboxylase (ACC) and CD36 to prevent fat buildup within the liver and prevent adipocytes from secreting too much leptin (96). Another study illustrated that *P. cyrtonema* polysaccharides could activate the T1R2/T1R3-mediated cAMP signaling pathway to stimulate GLP-1 production and decrease appetite (97). Furthermore, some investigations have demonstrated that the microbial fermentation of TCMPOs to form short-chain fatty acids (SCFAs) may encourage the production of GLP-1 and PYY (discussed in detail later), leading to elevated insulin and reduced glucagon release as well as appetite suppression (98, 99). The sole known orexigenic or hunger hormone, ghrelin, has been linked to the management of long-term energy balance as well as has the ability to increase appetite and meal initiation (4). Cholecystokinin (CCK), another anorexigenic intestinal hormone, is released in response to meal consumption and is involved in satiety (4). However, the potential effects of TCMPOs on ghrelin and CCK responses and the TCMPOs-mediated ghrelin/CCK relation to hunger and satiety ratings have rarely been reported and still require further research.

### Inhibition of fat absorption and synthesis

#### Impeding the digestion and absorption of exogenous lipids

It is common knowledge that obesity occurs when energy intake exceeds energy consumption; thus, it works well to prevent obesity by limiting the absorption of high-energy foods, especially fat from food. An essential lipolytic enzyme generated and released by the pancreas, pancreatic lipase (PL) is primarily involved in the hydrolysis of 50–70% of total dietary fats and is essential for the absorption of dietary triacylglycerols, which it does by hydrolyzing them into monoacylglycerols as well as fatty acids (6, 100–102). Emerging studies have found that TCMPOs could act as an effective inhibitor of PL to exert potentially antiobesogenic effects (Figure 2; Table 3). For example, Wu et al. (22) assessed the anti-pancreatic lipase activity *in vitro* about four polysaccharide fractions (DFP-H, DFP-M, DFP-U, and DFP-P) obtained from *D. indusiate* through different extraction techniques, as well as the inhibition of PL has been determined as 58.39 ± 0.98%, 52.65 ± 0.83%, 54.67 ± 1.03%, and 63.20 ± 1.11%, correspondingly. Other researches (103–107) also demonstrated that TCMPOs exhibit obvious inhibitory effects on PL, which indicates that TCMPOs could be developed as potential antiobesity agents to impede the digestion and absorption of dietary fat.

**TABLE 3:** Effects of TCMPOs on pancreatic lipase and bile acid micelles.

No.	Source	Compound fractions	Models	Methods	Daily intake and period	Inhibitory effects on lipase	Bile Acid-Binding Capacity	Others	References
1	*Plantago asiatica* L.	PLP	–	*In vitro*	–	At the PLP concentration of 10 mg/ml, the lipase activities were significantly reduced	The PLP at different concentrations (2.5 and 10 mg/ml) bound more BAs than that of simvastatin	At the concentrations of 2.5 and 10 mg/ml resulted in a significant (*p* < 0.05) reduction of α-amylase activity; At a level of 2.5 mg/ml or 10 mg/ml Significantly decreased the activity of pepsin; the PLP at 2.5 and 10 mg/ml affected the stability of the cholesterol-micelle and significantly (*p* < 0.05) reduced the amount of the unbroken cholesterol-micelle	(106)
2	*Plantago asiatica* L.	PLP	–	*In vitro*	–	–	PLP showed significant binding capacities against cholic and chenodeoxycholic acids	Exhibited scavenging abilities against hydroxyl, peroxyl anion, and DPPH radicals *in vitro*	(180)
3	*Laminaria japonica*	CGF-3	–	*In vitro*	–	The PL activity was significantly decreased with the increase concentration of CGF-3	–	–	(105)
		DSCGF-3	–	*In vitro*	–	Showed lower inhibitory activity on PL than that of CGF-3 at the same concentration	–	–	
4	*Auricularia auricula*	AAP	CED-mice	*In vivo*	120 mg/kg of BW for 8 weeks	–	–	The administration of AAP caused increasing LPL activity	(119)
5	*Cassia tora*	WSPs	–	*In vitro*	–	At the WSPs concentration of 80 mg/ml, the lipase activities were significantly reduced	WSPs could effectively bind BAs	High concentration of WSPs significantly increased the protease activity and decreased the amount of intact cholesterol micelle; had inhibitory effects on the activities of α-amylase	(103)
6	*Armillariella Mellea*	AAMP	HFD-mice	*In vivo*	50∼200 mg/kg of BW for 35 days	–	–	AAMP enhanced the level of LPL and the expressions of two critical lipases (adipose triglyceride lipase and hormone-sensitive lipase)	(123)
7	*Dictyophora indusiata*	DFP-H DFP-M DFP-U DFP-P	–	*In vitro*	–	At the concentration of 5.0 mg/mL, the inhibitions on the PL of DFP-H, DFP-M, DFP-U, and DFP-P were measured to be 58.39 ± 0.98%, 52.65 ± 0.83%, 54.67 ± 1.03%, and 63.20 ± 1.11%, respectively	Both DFP-P and DFP-H showed significantly higher BA-binding ability, respectively, followed by lower in DFP-M, and the lowest in DFP-U	DFP-P had the highest cholesterol-binding ability among all samples	(22)
8	*Stichopus variegatus*	FG dsFG crFG	–	*In vitro*	–	At the concentration of 0.50, 1.25 and 2.00 mg/mL, the PL activities were reduced by 16.9, 55.4, and 61.4% Showed lower inhibitory activity on PL than that of FG Showed lower inhibitory activity on PL than that of FG	–	FG dose-dependently inhibited the activity of α-amylase; Compared with that of FG, the inhibitory activity of dsFG and crFG on α-amylase was significantly decreased	(104)

PL, pancreatic lipase; BA, bile acid; LPL, lipoprotein lipase; HFD, high-fat diet; CED, cholesterol-enriched diet; – not detect.

Bile acids, another important physiological substance that is closely related to lipid metabolism, are the end products of cholesterol catabolism in the liver and secreted into the small intestine, which might activate PL and form micelles containing dietary fat and lipophilic vitamins (A, D, E, and K), thus facilitating fat absorption, distribution, metabolism, and excretion (108–110). Studies have shown that TCMPOs could bind with BAs (Figure 2; Table 3) and destroy the mixed micelles formed by BAs with dietary lipids, thus preventing the digestion and absorption of dietary lipids. Hu et al. (106) reported the BAs-binding abilities of *Plantago asiatica* L. polysaccharides, which could bind more BAs than simvastatin (a commercially available hypolipidemic drug) at experimental concentrations (2.5 and 10 mg/ml). Moreover, Shi et al. (111) observed that *Ophiopogon japonicus* polysaccharides (MDG-1) could reduce the synthesis of cholesterol in the liver and inhibit body growth within obese mice by binding to BAs in the lumen, reducing their reflux into the liver, and suppressing the enterohepatic circulation of BAs. In addition to the two principal targets of preventing fat digestion and absorption demonstrated above, the polysaccharides extracted from some kinds of TCM could also affect how well exogenous lipids are absorbed and digested by decreasing the activity of α-amylase, pepsin and trypsin (103, 104, 106, 107), affecting the stability of the cholesterol-micelle and reducing the amount of the unbroken cholesterol-micelle (22, 103, 106).

#### Weaken the synthesis of endogenous lipids

The size (hypertrophy) and quantity (hyperplasia) of adipocytes define the bulk of adipose tissue (112–114). The hypertrophy of adipocytes is mainly caused by lipid accumulation, and the hyperplasia of adipocytes is strongly linked to the growth as well as differentiation of preadipocytes (46, 112). Multiple reports have demonstrated that polysaccharides from diverse TCM sources may reduce the production of endogenous lipids by preventing preadipocyte differentiation and promoting the apoptosis of adipocytes (Figure 2) (46, 115, 116). For example, *Morus alba* L. polysaccharides (JS-MP-1) could decrease the cell viability of 3T3-L1 within a dose-dependent manner to 91, 75, 68, and 54% viabilities at the experimental levels of 50, 100, 200, and 500 μg/ml, correspondingly, suggesting that it might prevent preadipocytes from growing and proliferating (46). In addition, JS-MP-1 also induced apoptosis within 3T3-L1 preadipocyte cells by inducing mitochondrial dysfunction and activating apoptosis-related proteins, like caspase 9 and 3 and poly (ADP-ribose) polymerase (PARP) (46). *Poria cocos* polysaccharides (PCCPs) showed the ability to decrease 3T3-L1 cell viability and inhibit intracellular lipid accumulation at relatively high concentrations (117). Xu et al. (118) reported *L. barbarum* polysaccharides (LBPs) presented similar inhibiting impacts upon the division for 3T3-L1 cells, which might be attributed to the lowered expression of the differentiation-related genes peroxisome proliferator-activated receptor γ (PPARγ) and CCAAT/enhancer attaching protein α (C/EBPα) after LBP treatment.

Lipoprotein lipase (LPL), a glycoprotein distinguishes free fatty acids from triglycerides in chylomicrons (CMs) as well as very low-density lipoproteins (VLDLs) (119–121). Accumulating evidence has revealed that polysaccharides from various TCM sources can reduce lipid deposition by modulating the activity of LPL (119, 122, 123). Yang et al. (123) demonstrated that *Armillariella Mellea* polysaccharides (AAMPs) enhanced the level of LPL and the expression of two critical lipases (adipose triglyceride lipase and hormone-sensitive lipase) and specifically inhibited the expression of sterol regulatory element binding protein-1c (SREBP-1c), leading to a significant reduction in fat accumulation in the liver. Moreover, it is found that the administration of TCM polysaccharides could also inhibit the mRNA expression concentrations of lipogenesis-related enzyme genes (acetyl-CoA carboxylase 1, ACC1; fatty acid synthase, FAS; long-chain elongase, ELOVL 6 and diacylglycerol acyltransferase, DGAT) and elevate the mRNA expression for a lipolysis-related gene (carnitine palmitoyltransferase-1 alpha, CPT-1α) (18, 118, 124–127), thus weakening the lipogenic pathway and strengthening the fat decomposition pathway.

### Modification of the gut microbiota and their metabolites

#### Modification of the gut microbiota

The human intestinal cavity contains a variety of microorganisms, most notably bacteria. More and more researches are pointing to the resident microbiota’s critical roles in a range of physiological as well as pathological bodily processes (26, 114, 128). Plenty of research conducted in the last few decades have focused on the relationship between gut bacteria and obesity. Research has indicated that a rise within the ratio of *Firmicutes* to *Bacteroidetes* (F/B) was observed in obese individuals, and increased F/B ratio correlates with strengthened energy absorption capacity, which ultimately results in obesity (114, 129, 130). For example, by transplanting the faecal microbiota from adult female twin pairs that were discordant for obesity into germ-free mice, Ridaura et al. (131) discovered that metabolic abnormalities linked to obesity could be spread through uncultured faecal communities.

**FIGURE 3:**
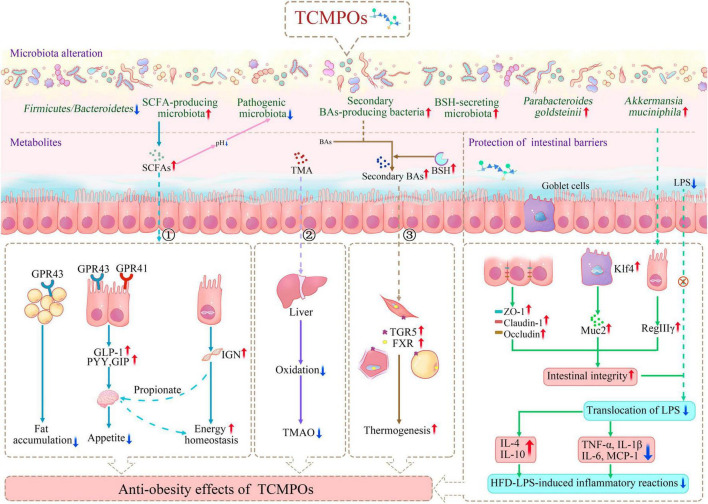
Antiobesity mechanism of TCMPOs based on the modulation of gut microbiota and their metabolites (① SCFAs; ② TMA; ③ Secondary BAs) and the protection of intestinal barriers. TCMPOs, traditional Chinese medicine-derived polysaccharides; GPR41(43), G-protein coupled receptors 41(43); GLP-1, glucagon-like peptide 1; PYY, peptide YY; SCFAs, short chain fatty acids; TMA, trimethylamine; TMAO, trimethylamine N-oxide; IGN, intestinal gluconeogenesis; BAs, bile acids; TGR5, takeda G protein Receptor 5; FXR, farnesoid X Receptor; BSH, bile salt hydrolase; ZO-1, zonula occludens-1; Klf4, kruppel-like factor 4; Muc2, mucin 2; LPS, lipopolysaccharide; RegIIIγ, regenerating islet-derived protein 3 γ; IL-4, interleukin-4; IL-10, interleukin-10; TNF-α, tumor necrosis factor-α; IL-1β, interleukin-1β; IL-6, interleukin-6; MCP-1, monocyte chemoattractant protein-1.

It has been discovered that some TCM sources include polysaccharides that can fight obesity through altering the gut microbiome (Figure 3; Table 4). Typically, for instance, *Stichopus japonicus* polysaccharides (SCSPs) and depolymerized SCSPs (d-SCSPs) exhibited antiobesity impacts within HFD-fed mice through changing the proportion of F/B; enhancing the abundances of *Akkermansia muciniphila*, *Parabacteroides goldsteinii* as well as SCFA-producing microbiota (*Bacteroides*, *Alloprevotella*, *Ruminiclostridium*, and *Butyricicoccus*); and decreasing the abundances of *Proteobacteria* and pathogenic *Escherichia-Shigella* (132–136). Furthermore, it was shown that purified *L. japonica* polysaccharide (LJP61A) may also significantly reverse the increased F/B ratio and deceased abundance of *Akkermansia* induced by the HFD-diet (137). *A. muciniphila*, a mucin-degrading bacterium commonly found in the gut, was found to have close ties to intestinal barrier integrity as well as resistant to insulin, which helps it fight obesity and related metabolic illnesses (138–140). In addition, the increasing abundance of *P. goldsteinii* was found to be mostly responsible for the anti-obesity benefits of *H. sinensis* polysaccharides, which was linked to improved intestinal integrity, decreased inflammatory levels, as well as improved adipose tissue thermogenesis (21). Overall, TCMPOs-dietary interventions were reported to combat obesity by regulating the ratio of F/B, boosting the colonization of some advantageous bacteria and decreasing the survival of detrimental bacteria linked to inflammation and the onset of obesity.

**TABLE 4:** TCMPOs improve the gut microbiota and intestinal barrier and alleviate inflammation.

	Source	Compound fractions	Models	Gut microbiota	SCFAs	Impacts on the intestinal barrier and Inflammatory Cytokines	Endotoxin	References
1	*Laminaria japonica*	LJP61A	HFD-fed C57BL/6 mice	↓F/B ↑*Akkermansia* spp.	–	↑ZO-1, occludin, goblet cells, Muc2, Klf4 ↓TNF-α,IL-6 and IL-1β↑intestinal integrity	↓LPS	(137)
2	*Stichopus japonicus*	d-SCSP, SCSP	HFD-fed mice	↓F/B ↑ *Akkermansia muciniphila, Parabacteroides goldsteinii, Bacteroides, Alloprevotella* ↓*Proteobacteria, Escherichia-Shigella*	↑Acetic acid ↑propionic acid ↑butyric acid	↓IL-6, IL-1β,MCP-1, TNF-α↑intestinal integrity	↓LPS ↓LBP	(134)
3	*Ganoderma lucidum*	WEGL	HFD-fed mice	↓F/B ↑*Bacteroides, Eubacterium spp, Roseburia spp.* ↓*Oscillibacter* spp, E. *fergusonii. Mucispirillum* spp.	–	↑ZO-1, occludin ↑intestinal integrity	↓LPS	(18)
4	*Ophiopogon japonicus*	MDG-1	HFD-fed C57BL/6 mice	↓F/B	↑Vinyl acetic acid ↑butyric acid	–	–	(141)
5	*Sargassum fusiforme*	SFF	HFD-fed mice	↓F/B ↑*Bacteroides*, *Lactobacillus*, *Alistipes* ↓*Helicobacter*	–	↑Zo-1 and Occludin-1 ↓IL-6, TNF-α, and IL-1β↑intestinal integrity	↓LPS	(30)
6	*Poria cocos*	WIP	ob/ob mice	↑*Lachnospiraceae, Alloprevotella, Clostridium IV, Parabacteroides, Ruminococcus, Bacteroides* ↓*Megamonas, Proteus*	↑Butyrate	↑Muc5, ZO-1, Occludin ↓TNF-α↑intestinal integrity	↓LPS	(32)
7	*Hirsutella sinensis*	H1	HFD-fed mice	↑*Parabacteroides goldsteinii, Flintibacter butyricus, Intestinimonas butyriciproducens, Clostridium cocleatum, Clostridium viride and Anaerotruncus colihominis* ↓*Pseudomonas aeruginosa, Escherichia coli and Shewanella algae*	↑SCFAs	↑ZO-1 ↑IL-10 (anti-inflammatory) ↓IL-1β, TNF-α (pro-inflammatory) ↓intestinal permeability	↓LPS	(21)
8	*Holothuria Leucospilota*	HLP	HFD-fed rats	↑*Bacteroides, Oscillospira*	↑Total SCFAs ↑acetic acid ↑propionic acid ↑butyric acid	↑IL-10 ↓IL-6, IL-12, TNF-α	–	(96)
9	*Dictyophora indusiata*	DIP	HFD-fed BALB/c mice	↓F/B ↓*Bacilli, Gammaproteobacteria* ↑*Bacteroidia, Lactobacillaceae, Ruminococcaceae*	↑Butyrate	↑Claudin-1, Occludin, ZO-1, Muc2, goblet cells ↑IL-4, IL-10 ↓TNF-α, IL-1b, IL-6, IL-1β MCP-1 ↑intestinal integrity	↓LPS	(161, 162)
10	*Polygonatum kingianum*	PSF	HFD-fed rats	↓F/B ↓*Lactobacillus*, *Psychrobacter* ↑*Bacteroides*, *Oscillibacter*	↑Total SCFAs ↑acetic acid ↑propionic acid ↑butyric acid	↑Occludin, ZO-1	↓LPS	(73)

HFD, high-fat diet; F/B, Firmicutes to Bacteroidetes; SCFAs, short chain fatty acids; Klf4, kruppel-like factor 4; ZO-1, zonula occludens-1; Muc2, mucin 2; Muc-5, mucin 5; IL-1b, interleukin-1b; IL-4, interleukin-4; IL-10, interleukin-10; TNF-α, tumor necrosis factor-α; IL-1β, interleukin-1β; IL-6, interleukin-6; MCP-1, monocyte chemoattractant protein-1. LPS, lipopolysaccharide; ↑, no significant difference; ↑or↓, increase or decrease significantly; – not detect.

#### Modification of microbially derived metabolites

Numerous routes may be involved in how TCMPOs impact the relationship between the host’s gut flora and itself. First, as shown in Figure 3 and Table 4, microbial fermentation of TCMPOs in the intestine enhances the synthesis of metabolites like SCFAs, consisting predominantly acetate, propionate, as well as butyrate, which are essential for the health homeostasis and wellbeing of the host. TCMPOs intervention could lead to an increased production of SCFAs and a lower luminal pH, which effectively inhibits the growth of pathogenic microbiota (134, 141). SCFAs were also reported as ligands for G-protein coupled receptors (GPCRs), GPR41 and GPR43, and propionate and butyrate showed closer affinity for GPR41, which serves as gut microbe-related energy sensors within the intestines and sympathetic nervous system (142–145). Propionate and acetate were reported to be more active in activating GPR43, which prevents fat from accumulating within adipose tissue, inhibits insulin signaling within adipocytes, and increases the metabolism of glucose and unincorporated lipids within other tissues (like liver and muscle) (59, 145, 146). For instance, treatment with *Morus alba.* L leaf polysaccharide (MLP) significantly reduced body weight gain and in a dose-dependent manner reversed the decrease in SCFAs concentration in HFD-fed mice (84). *Astragalus* polysaccharides (APS) could ameliorate hepatic lipid metabolism within HFD-fed mice by enriching the abundance of a potent acetate-producing bacterium (*Desulfovibrio vulgaris*) (147). Moreover, SCFA-mediated GPCR activation in the gut could induce the secretion of the endocrine hormones GLP-1, PYY, and glucose-dependent insulinotropic polypeptide (GIP), which have been proven to be important in preventing or treating obesity (144, 148). Additionally, by inducing intestinal gluconeogenesis (IGN) gene expression in enterocytes, SCFAs derived from the microbial fermentation of polysaccharides might enhance a number of aspects of energy metabolism in both insulin-sensitive and insensitive states (149, 150). Propionate, being an IGN substrate, can typically directly start a gut-brain neuronal circuit that has positive effects on energy management and glucose regulation (149, 150). In summary, TCMPOs exhibit an antiobesity effect by increasing the production of SCFAs, which could decrease the pH of the lumen, activate GPR41 and GPR43, induce the secretion of certain endocrine hormones, and activate IGN to improve energy metabolism.

In addition to SCFAs, intestinal microorganisms can also generate trimethylamine (TMA), which may affect host metabolism (Figure 3). The intestinal anaerobic bacteria can metabolize choline as well as L-carnitine from food sources (e.g., animal liver, red meat, egg yolks and fish) to create TMA (151). This gut-microbially produced TMA can be further decomposed into dimethylamine (DMA) and methylamine (MA) or it can be further carried into the liver and subsequently turned into trimethylamine N-oxide (TMAO), which is linked to an increased risk of obesity (151–153). Chen et al. (154) demonstrated that *Flos Lonicera* polysaccharide supplementation could reverse the increase in TMAO level caused by an HFD. Similarly, after LBP treatment, the level of plasma TMAO was considerably lower within the HFPD (LBP + HFD-diet) group compared with the HFD group (155). These investigations showed that the polysaccharides of multiple TCMs could lessen the oxidation of TMA and thereby serve as a possible dietary supplement to help reduce obesity and related diseases via gut-organ axes.

Aside from generating novel metabolites, intestinal bacteria may also modify the physicochemical properties of endogenous metabolites (Figure 3). BAs, as mentioned above, are small molecules that have important functions in controlling lipid metabolism. Ninety-five percent of BAs are reabsorbed efficiently from the distal ileum, entering the enterohepatic circulation, and are circulated back to the liver (151). In addition, BAs can also be converted from primary to secondary BAs in the colon, a function that is restricted to a much narrower range of anaerobic bacteria (typically *clostridial* species) (156). Both primary and secondary BAs are essential signaling molecules that can modify energy metabolism through interact with receptors, Takeda G protein Receptor 5 (TGR5) (157, 158), and Farnesoid X Receptor (FXR) (6, 159). Activation of TGR5 and FXR facilitates energy expenditure and encourage thermogenesis, especially in the liver, muscles and brown adipose tissue (BAT), which lowers body weight (151, 156, 158). Studies have reported that TCMPOs supplementation (such as supplementation of PCCPs and *H. sinensis* polysaccharides) raises the abundance of secondary BA-producing bacteria (*Clostridium*), thus enabling the better activation of TGR5 and FXR, which may serve as a potential antiobesity mechanism for TCMPOs (21, 32). Moreover, HFD-induced dysbiosis can lead to the functional loss of bile salt hydrolase (BSH), which in the gut microbiota is in charge of deconjugating primary conjugated BAs, resulting in an imbalance in primary and secondary BAs in the colon, thus inducing glycolipid metabolism-related diseases (151, 158). Huang et al. (160) observed that *Gracilaria lemaneiformis* polysaccharide intervention markedly raised the proportional abundances of BSH-secreting microbiota (*Bacteroides* and *Lactobacillus*), hence encouraging the conversion of primary bile acids into secondary bile acids and the excretion of secondary bile acids, which in turn lowers cholesterol and helps prevent fat buildup brought on by a high-fat diet. Overall, fermentable TCMPOs could fight against obesity by generating SCFAs and TMA/TMAO and modifying the physicochemical properties of BAs. In addition, other microbially derived metabolites, such as branched-chain amino acids (BCAAs) and serotonin (5-HT), have also been found to be strongly associated with the emergence of obesity (151). Future studies on how TCMPOs affect the production of BCAAs and 5-HT should be conducted.

### Protection of intestinal barriers

Numerous studies point to the interaction among intestinal permeability as well as TCMPOs as one mechanism connecting obesity and related diseases (Figure 3; Table 4). Oral treatment with TCMPOs beneficially maintained intestinal barrier integrity primarily by promoting intercellular tight junctions and strengthening the intestinal mucous layer. As shown in Figure 3 and Table 4, various studies have reported that TCMPOs, such as *L. japonica* polysaccharides and *S. fusiforme* polysaccharides, could elevate the expression of tight junction proteins (TJPs), including zonula occludens-1 (ZO-1), claudin-1 and occludin, to regulate the tight junction structure, thus protecting the integrity of the gut epithelium (18, 30, 32, 161, 162). Additionally, *L. japonica* polysaccharide (LJP61A) administration enhanced the mRNA levels of Kruppel-like factor 4 (Klf4, a marker of goblet cells) and mucin 2 (Muc2, a protein secreted by goblet cells) in HFD-fed mice, indicating that LJP61A may be able to counteract the HFD-induced reduction in goblet cells (137). Specifically, it has been reported that LJP61A may also elevate the relative abundance of *A. muciniphila*, which is reported that it may enhance the expression of regenerating islet-derived protein 3 γ (RegIIIγ) in response to gram-positive bacteria in the intestine and aid in its own survival, thereby promoting the development of the intestinal mucus layer (137, 163). Lipopolysaccharide (LPS), a cell wall component of gram-negative pathogenic bacteria, can enter the circulation and trigger a variety of transcription factors associated with inflammation, resulting in the initiation of chronic low-grade inflammation that is known as a causal factor of obesity and hepatic steatosis (32, 164). Oral treatment with TCMPOs could improve the intestinal barrier to prevent LPS from entering the circulation, hence attenuating HFD-LPS-induced inflammatory reactions and exerting the antiobesogenic effects of TCMPOs. For example, *D. indusiata* polysaccharides (DIPs) could maintain intestinal integrity and alleviate HFD-induced obesity by significantly reducing the levels of LPS and proinflammatory cytokines (tumor necrosis factor-α, TNF-α; interleukin-1β, IL-1β; interleukin-6, IL-6; monocyte chemoattractant protein-1, MCP-1) and enhancing the secretion of anti-inflammatory cytokines (interleukin-4, IL-4; interleukin-10, IL-10) (161, 162). It is worth noting that SCFAs, generated by microbial fermentation of TCMPOs, are considered vital energy sources for intestinal epithelial cells and can promote the development of the intestinal barrier (165).

## Conclusion and future perspectives

Traditional Chinese medicine-derived polysaccharides, acting as important components with few adverse reactions and significant biological activity, have gained increasing attention during the last decade. In this review, the various extraction and purification methods, structure-activity relationship and antiobesity mechanisms of TCMPOs were systematically summarized. In particular, the antiobesity effects of TCMPOs were shown via mechanisms including the modulation of appetite and satiety, inhibition of fat absorption, modification of the gut microbiota and their metabolites as well as the protection of intestinal barriers. Furthermore, the relationship between TCMPOs’ antiobesity actions and structure is emphasized. However, there are many questions yet to be noted. (1) It is imperative to develop novel extraction technologies to improve the extraction efficiency due to the limitations of the thermal-assisted extraction of TCMPOs mentioned above. In addition, the structure of TCMPOs varies with geographical origin, the collected part of the herb, the harvesting season, and the processing method. It is unreliable to obtain TCMPOs from different sources of TCM with the same extraction method. Therefore, future research should screen out specific extraction methods based on the structural characteristics of various TCMPOs to protect the structural integrity of the target polysaccharides. (2) Consistency, repeatability, and reliability of the results are hard to maintain due to different testing circumstances and the varied qualities of raw materials. It is necessary to establish standardized procedures for the gathering and processing of the source material as well as for the extraction, isolation, and purification of TCMPOs. (3) Owing to TCMPOs’ extensive structural diversity and complexity, the senior structural characteristics have not been clarified, and the relationships between their senior structure and antiobesity activity have not been established. Therefore, we need to learn from the structural identification methods of other biomacromolecules to accelerate the structural analysis of TCMPOs. Based on the accurate structure of TCMPOs, further research and confirmation should focus on the structure-bioactivity interaction of TCMPOs to better clarify the structural basis of their antiobesogenic activity. (4) Given that obesity and associated metabolic disorders are often multigene variant, multitarget interfered and multidisease related, it is inappropriate to treat obesity in a single way, while TCMPOs possess the advantages of having multiple targets, being less toxic and having side effects in the treatment of obesity than current therapeutics. However, the feature structure and functional groups within TCMPOs that exert antiobesogenic activity, as well as the antiobesity mechanism of TCMPOs, have not been systematically clarified. Thus, there is a great need for further clarifying the feature structure in TCMPOs and its molecular targets responsible for the observed antiobesogenic activity. (5) Although the antiobesogenic activity of TCMPOs has been demonstrated in many experiments, more attention should be given to clinical studies because animal models or human body tissues and cell experiments *in vitro* do not accurately represent their existence in the human body.

This review may serve as a valuable reference to the extraction, purification, structural-property correlations and antiobesity mechanism of TCMPOs while offering a possible foundation for their usage to the food along with medical fields. Future studies should concentrate on resolving the aforementioned issues, and therapeutic approaches utilizing TCMPOs for the management of obesity and obesity-related disorders could represent yet another advancement in the realms of functional foods, pharmaceuticals, and medicine.

## Author contributions

NZ: Investigation, Methodology, Writing—original draft, Writing—review and editing, Project administration. XC: Funding acquisition, Investigation, Methodology, Supervision, Writing—original draft. XW: Writing—original draft. JG: Investigation, Methodology, Writing—review and editing. JC: Investigation, Writing—review and editing. SG: Investigation, Writing—review and editing, Funding acquisition, Methodology, Project administration, Resources, Supervision.
